# Diagnostic approach in cases with suspected work-related asthma

**DOI:** 10.1186/1745-6673-8-17

**Published:** 2013-06-14

**Authors:** Tor B Aasen, P Sherwood Burge, Paul K Henneberger, Vivi Schlünssen, Xaver Baur

**Affiliations:** 1 Department of Occupational Medicine, Haukeland University Hospital, NO-5021 Bergen, Norway; 2 Birmingham Heartlands Hospital, Birmingham, UK; 3 Division of Respiratory Disease Studies, National Institute for Occupational Safety and Health, Morgantown, WV MS 2800, USA; 4 Department of Public Health, Section of Environment, Occupation and Health, Danish Ramazzini Centre, Aarhus University, Aarhus, Denmark; 5 Institute for Occupational Medicine, Charité University Medicine, Berlin, Germany

**Keywords:** Asthma, Occupational disease, Diagnosis, Work-related

## Abstract

**Background:**

Work-related asthma (WRA) is a major cause of respiratory disease in modern societies. The diagnosis and consequently an opportunity for prevention are often missed in practice.

**Methods:**

Based on recent studies and systematic reviews of the literature methods for detection of WRA and identification of specific causes of allergic WRA are discussed.

**Results and Conclusions:**

All workers should be asked whether symptoms improve on days away from work or on holidays. Positive answers should lead to further investigation. Spirometry and non-specific bronchial responsiveness should be measured, but carefully performed and validly analysed serial peak expiratory flow or forced expiratory volume in one second (FEV_1_) measurements are more specific and confirm occupational asthma in about 82% of those still exposed to the causative agent. Skin prick testing or specific immunoglobulin E assays are useful to document allergy to high molecular weight allergens. Specific inhalational challenge tests come closest to a gold standard test, but lack standardisation, availability and sensitivity. Supervised workplace challenges can be used when specific challenges are unavailable or the results non-diagnostic, but methodology lacks standardisation. Finally, if the diagnosis remains unclear a follow-up with serial measurements of FEV_1_ and non-specific bronchial hyperresponsiveness should detect those likely to develop permanent impairment from their occupational exposures.

## Background

At least 15% of adult asthma is induced or triggered by factors in the workplace [1]. Early diagnosis can improve the prognosis of work-related asthma since cessation of exposure after appearance of asthmatic symptoms and identification of specific sensitization within the first months after onset of symptoms, may permit a full recovery. In addition, diagnosis of a case of work-related asthma (WRA) is a sentinel event that should lead to effective primary preventive measures in the individual workplace or in a branch of industry [2-4]. The reader is referred to recent comprehensive and systematic literature reviews [5-11]. Diagnosis of work-related asthma is in many cases a complex undertaking consisting of various diagnostic tests and procedures. The main object of diagnosis is to identify the cause of the asthma, in particular to separate occupational asthma from asthma unrelated to work, assisting decisions about intervention to prevent further exposure to the patient and is also important for handling compensation claims. The possibilities for prevention and degree of economic support for the patients may differ between industries and countries. The optimal diagnostic strategy will vary depending on medical as well as economic and juridical evidence.

Obvious preconditions for considering a diagnosis of WRA are typical diagnostic findings of asthma (see below) and an association of asthma symptoms with exposure at work. Asthma present before occupational exposure but associated with worsening at the start of a new occupational exposure indicates a diagnosis of work-aggravated asthma (WAA). Asthma relapsing or becoming more severe some time after exposure to a new occupational allergen may be due to occupational asthma, and does not preclude the diagnosis. A thorough clinical and occupational history should be obtained in all cases [12,13].

The diagnosis of WRA can be performed in three steps:

1. Making the diagnosis of asthma.

Asthma diagnosis is performed according to recent international guidelines [14,15]. However, the diagnosis of asthma is not always straightforward. Asthma is a heterogeneous disease, and the diagnostic criteria are ill-defined and vary between studies [16,17]. Further, there is considerable overlap between asthma and chronic obstructive pulmonary disease (COPD) [18]. A doctor’s diagnosis of asthma may be frequently questionable [19].

2. Identification of the workplace as the cause of the patient’s asthma.

Objective verification of the association between work exposure and airflow limitation is a basis for diagnosing WRA, both for new onset occupational asthma (OA) and WAA.

3. Identification of a specific agent causing WRA.

This step is most demanding on resources. A specific diagnosis is needed before appropriate remedial action can be taken in the workplace, but the full diagnostic armamentarium is only available at few centres worldwide [20]. The extent of diagnostic investigation will depend on what resources are available and on a decision analysis that takes into account the consequences of false positive and false negative diagnoses [21].

## Diagnosis of allergic work-related asthma

Recognized exposure to a known allergen at work and a provisional diagnosis of WRA should lead to an extensive diagnostic work-up to identify the agent and objectively confirm its causal role. Occupational allergens conform to the general definition of allergens [22].

To reach the diagnosis of WRA several pieces of evidence are necessary:

• Objective information of exposure to known sensitizers.

• Demonstration of an association between exposure at work and airflow limitation by lung function testing or changes in non-specific bronchial hyperresponsiveness (NSBHR) or changes of airway inflammation (e.g. sputum eosinophils).

• Identification of a specific allergic reaction to the occupational agent (rather than an acute irritant reaction).

Note: There are many OA-inducing agents and worksites generally included in the lists of airway allergens and irritants (for details see latest summaries [23,24] and Irritants: Evidence table of reporting OA or occupational COPD due to irritants (Authors: X. Baur, P. Bakehe, H. Vellguth, JOMT), ACGIH Tables: Occupational agents with respiratory effects according to ACGIH and/or classified with the H334 (R42) phrase and/or H335 (R37) phrase plus EU regulations (Author: X. Baur); however, due to negative allergological findings for many of them pathogenetic mechanisms are still unknown; examples include most cases of isocyanate and potroom asthma [25].

Diagnostic tests can be divided into those which should be available to all respiratory physicians and those generally confined to specialist in occupational lung diseases.

### Diagnostic methods suitable for all respiratory and occupational physicians

All working patients with asthma and COPD should be asked whether their symptoms improve on days away from work or on holidays. Further investigation is required for all positive respondents.

**Questionnaires**. Questionnaires are extensively used as screening tools in epidemiological studies. Several epidemiological questionnaires are in current use [26-28]. Questions about work exposure and association of symptoms to work may be used in clinical diagnosis.In the clinical setting questionnaires that identify symptoms of wheeze and/or shortness of breath which improve on days away from work or on holiday have a high sensitivity, but relatively low specificity for OA [28-34].

**Medical histories by experts**. There is general agreement that medical histories taken by experts have high sensitivity, but their specificity may be lower [28,29,32,35-38].

**Exposure assessment**[39]. This comes both within the compass of a generalist and a specialist. A generalist should elicit exposures in high-risk occupations, in which asthma should be assumed to be occupational unless excluded by objective tests. *The workers reported from population studies to be at increased risk of developing asthma include; bakers, chemical workers, cleaners, cooks, electrical and electronic production workers, farm workers, food processors, forestry workers, healthcare workers, laboratory technicians, mechanics, metal workers, painters, plastics and rubber workers, storage workers, textile workers, waiters, welders and wood workers*. A specialist should identify specific exposures, with a systematic occupational history, scrutiny of exposure documentation such as material safety data sheets (MSDSs), internal reports and industrial hygiene measurements from the industry. The failure to find a sensitiser on a MSDS should not preclude the diagnosis of occupational asthma, as many sensitisers are not regularly listed, particularly those in low concentration, those that are only present in certain circumstances, such as when heated and those given non-specific titles such as preservative, biocide, fragrance, resin etc.

**Spirometry**[40,41]. Spirometry is required in all patients considered for WRA. It is used as a main instrument for monitoring lung function longitudinally during surveillance, also during measurement of non-specific bronchial hyperresponsiveness (NSBHR) and in specific inhalation challenge. Many workers with WRA have normal spirometry when seen in a clinic. There is also a role for measurement of airway resistance or specific conductance to monitor lung function when a patient cannot record FEV_1_ reliably.

**Pre- to post-shift changes in lung function** cannot be recommended for the validation or exclusion of WRA [42,43]. Serial PEF and spirometry measurements have been shown to be superior to cross-shift change in diagnosing WRA [43].

**Measurement of non-specific airway responsiveness**[44,45]. Challenge with methacholine is part of the initial diagnostic work-up. It can also be measured before and after a period of work exposure and during SIC. Normal NSBHR does not exclude OA.A large number of concordant studies from different centres using different methodologies demonstrated that increased NSBHR is often found in workers with WRA. There are, however, many reports of normal methacholine or histamine reactivity within 24 hours of exposure in workers with confirmed OA [28,29,32,34,36,38,46-57].

**Serial lung function testing**. If clinical OA exists, exposure to routine levels of the causative agent should result in a measureable decline in lung function in the hours after exposure. This is usually performed by a simple expiratory peak flow (PEF) meter or by a portable spirometer [58]. Measuring PEF or FEV_1_ during periods of work and away from work may document a causal relationship between exposure and airflow reduction [58]. Technical issues related to interpretation have been extensively studied [59]. The frequency and duration of the recording depends on the method of analysis, but in general, at least 4 readings/day are required, although 8 readings a day allows shorter records, with usual exposure on work days and no changes in treatment on days away from work. A 3-week record containing at least 3 periods off work would be suitable for most forms of analysis [60-62]. The sensitivity and specificity of serial peak flow measurements performed after extensive instruction to patients and using quality control are high in the diagnosis of OA, for which there is a recent systematic review with a pooled sensitivity of 82% and specificity of 88%. Peak flow measurements require encouragement and instruction of the patient; otherwise at least one third of the subjects will not produce reliable data [30,31,43,48,56,62-68].Note: New handheld electronic spirometers storing the whole flow volume curves and automatically checking for reproducibility and other quality criteria (e.g. ATS/ERS 2005) will allow easy quality control and provide additional parameters [41]. Their practicability, including the analysis of the large data set that can result from testing one patient, remains to be shown.

### Allergological tests

• The skin prick test (SPT) is the mainstay of allergological testing. SPT is generally regarded as sensitive, but with less specificity. The test should be performed according to international guidelines [69]. Standardized material is rarely available in suspected occupational allergy cases, but crude extracts produced locally may be useful. SPT is usually not performed in low molecular weight (LMW) allergy (with the exception of platinum salts and reactive dyes).

•Specific immunoglobulin E (IgE) tests are often less sensitive, but possibly more specific than SPT. A major shortcoming of allergological tests is the lack of standardization of most occupational allergen extracts as well as of the antibody measuring assays [70-72].

•Both SPT and specific IgE tests are in general sufficiently sensitive for detecting type I sensitisation and OA caused by most high molecular weight agents (HMW), but are not specific for diagnosing asthma [34,73].

•Both SPT and specific IgE tests are sensitive for detecting type I sensitisation and occupational asthma caused by acid anhydrides and some reactive dyes, but have a lower specificity for diagnosing asthma in these cases [74-78]. Skin prick tests are highly sensitive but less specific for occupational asthma caused by complex platinum salts [53,54].

•Other in vitro studies such as mediator release test (i.e. of histamine) in an allergen-antibody reaction and release of cytokines during in vitro stimulation [79] are, to date, not routine tests.

### Diagnostic methods suitable for specialists in occupational lung diseases

Specific inhalation challenge tests (SIC) [80,81]. SIC are commonly regarded as a reference method for diagnosing sensitizer-induced WRA. However, the test is in practice not well standardized internationally and is sparsely available [82]. They are subject of a current ERS taskforce whose preliminary indications are:

• Confirming the diagnosis and cause of OA when other objective methods are not feasible, have failed or provide indeterminate results.

• As a rapid way to evaluate the possibility of OA caused by a sensitizing agent when the net potential clinical benefit of the test is expected to exceed the risk of complications.

• When a new (not formerly described) specific cause of OA is suspected.

• For research purposes.

Methods for performance of specific inhalation tests:

These may be performed in a specially designed laboratory in two main ways:

1. Controlled laboratory challenge.

By use of this method controlled concentrations of the suspected sensitizers (standardized allergen extract) are administered in controlled concentration and dose either by the inhalation chamber method or by closed-circuit methodology [81].

Laboratory-based specific challenge testing may produce false negative results, which has been shown when comparing laboratory challenge with workplace challenges which confirmed occupational asthma [83].

2. Workplace challenge test or realistic challenge.

This is an attempt to reproduce actual work processes and exposures which are performed in line with the Jack Pepys tradition [84].

The aim of a workplace challenge is to make supervised lung function similar to a bronchial provocation testing in the workplace after suitable control measurements have been made without exposure in the workplace on separate days. Measurements of NSBHR, cells in induced sputum, fractional exhaled nitric oxide (FeNO) etc. can be made in conjunction with the workplace challenge. The patient should be exposed to the usual concentrations of the potential causative agent during the challenge that may last up to 2(−4) hours of usual exposure. It is important to ensure that the usual work practices are taking place during the workplace challenge which may not always be easy to achieve.

Safety considerations are important, and many workplaces do not provide clean and safe environments for medical treatment and the whole site may be the cause of the asthma. In these circumstances any emergency treatment of a severe asthmatic attack would involve removing the worker from the workplace and often from sources of electrical supply, typically needed to perform spirometry and operate nebulizers.

Limitations of specific inhalation challenge

Specific bronchial provocation testing can produce false positive and false negative results. It is particularly difficult to set up when the exposures are complex (such as with welding fume or metal working fluid exposures) or when the process being mimicked involves high temperatures, explosive or inflammable material. SIC is in general poorly standardized. Even if the procedure is usually regarded as the gold standard for diagnosing WRA, it requires much experience to adequately define the challenge procedure and to know which parameter to use when assessing the sensitivity and specificity of this test.

The earliest attempt at assessing the validity of SIC used continued work with exposure to the original suspected causative agent without reduction of exposure for a year. The subsequent absence of deterioration in asthma was taken as an indicator of no WRA, and the need for removal from exposure because of repeated severe asthmatic attacks indicated OA. Using these two criteria, the sensitivity for specific bronchial provocation testing with isocyanates up to a 30 minute exposure to 0.02 ppm was 82% and for colophony up to a 15 minute exposure to heating neat colophony was 100%. The relevant specificities were 67% for colophony and 100% for isocyanate exposure [85].

More recently Rioux [83] performed work place challenges in 99 workers who had negative specific bronchial provocation testing but had a good history of OA. Twenty-nine of these had a positive workplace challenge, some with subsequent positive bronchial provocation testing when different agents were tested. Of those who had negative workplace challenges, 34/70 had asthma or rhinitis excluded following further investigation. False negative specific challenges were therefore found in 29/65 with asthma or rhinitis and a good history of workplace deterioration [83].

There are no studies evaluating the criteria for a positive or negative workplace challenge. However, the criteria for detecting late asthmatic reactions from specific challenges proposed by Stenton and colleagues [86] are probably appropriate. These criteria specify at least three control days without exposure, calculating the pooled standard deviation for the FEV_1_ measurements on the unexposed days and requiring at least 2 consecutive measurements on the workplace challenge day to be below these pooled standard deviation measurements [86]. This method has been applied to unsupervised serial measurement of PEF and found to have a specificity of over 90% with specific challenge testing as the standard [87].

In summary, in spite of their limitations carefully controlled specific challenges come closest to a gold standard test for some agents causing OA [42,43,49,52]. Further, a negative test in a worker with otherwise good evidence of OA is not sufficient to preclude the diagnosis [42,43,49,52].

### Non-invasive methods to assess airway inflammation

Several non invasive methods have been used to assess and monitor airway inflammation in asthma in the last decade.

Three of these methods have been standardized by international committees:

• induced sputum

• exhaled nitric oxide (FeNO)

• exhaled breath condensate (EBC).

Analysis of induced sputum is a valid and reproducible method for studying airway inflammation [88,89]. The method consists of inducing sputum production by having the patient inhale a hypertonic saline solution. Sputum is subsequently processed and cytospins are prepared and stained. In addition, inflammatory mediators can be measured in sputum supernatant [89]. The count of eosinophils is the most repeatable parameter in induced sputum and is a reasonably good non-invasive index of airway eosinophilia since there is fairly good agreement between the evaluations of eosinophils in induced sputum, in bronchial biopsy specimens, and in bronchoalveolar lavage [90]. The validity of this method is attested to by several studies, which have shown that sputum inflammatory indices such as eosinophils and eosinophil cationic protein (ECP) are increased by exposure to common allergens and are reduced with corticosteroids [91]. There is evidence that induced sputum may be useful in clinical practice since evaluation of sputum eosinophils was shown to be equivalent to NSBHR for discriminating asthma from other conditions, and assessment of sputum eosinophilia improved the management of asthma [17,92,93].

Several studies reported sputum eosinophilia in both early and late reactors between 6 and 24 hours after SIC in OA caused by both high- and low-molecular weight occupational agents. Moreover, the addition of sputum cell counts to monitoring of PEF increased the specificity of this test suggesting that monitoring airway eosinophils in induced sputum improves the diagnosis of OA [94-96]. Indeed, induced sputum may be useful in the diagnosis and follow-up of subjects with WRA [97]. Its utility in epidemiological studies has not been evaluated.

Eosinophil counts in sputum may have other diagnostic uses. Sputum might be helpful in differentiation between WAA and OA due to a workplace sensitiser that is superimposed on existing asthma, since two studies have shown that exposure to occupational agents in asthmatics not sensitised to the agents did not induce airway inflammation and did not change the sputum cell composition [88,98]. The analysis of induced sputum has also been found to be useful in the identification of occupational eosinophilic bronchitis. This condition is characterized by cough on exposure to occupational agents, without any change in lung function tests [99].

Sputum eosinophilia is not present in all workers with allergic WRA. Compared with those with sputum eosinophilia, those without eosinophilia were exposed to similar agents, had similar changes in lung function related to work exposure but had less NSBHR [46].

Inhalation of hypertonic saline is not completely non-invasive, and can cause bronchoconstriction in subjects with hyperresponsive airways. Its success rate is less than 100%, since some subjects are unable to produce adequate samples. In addition, the induction is time-consuming and processing the samples is laborious and needs to be performed soon after collection [89]. The conclusions from the ERS evidence-based guidelines was [10];

• Sputum eosinophils increasing by >1% post SIC or workplace exposure may support a diagnosis of OA when the FEV_1_ has fallen <20%.

• The presence or absence of increased sputum eosinophils is not useful in selecting or excluding those who might have work-related asthma.

Exhaled nitric oxide (FeNO) is a non-invasive tool for assessing airway inflammation in asthma [100]. NO is produced by various cells in the lung either resident or recruited during the inflammatory process. Exhaled NO is generally measured on line, the subject blowing directly into the analyser with immediate results. Portable analysers are also available. It is also possible to have a remote breath collection into inert bags, with subsequent analysis. NO concentration is increased in the exhaled air from patients with asthma and decreased by corticosteroid therapy [101-103]. There is a positive correlation between FeNO and sputum eosinophils in asthma [104]. In clinical practice, evaluation of FeNO was equivalent to sputum eosinophils in diagnosing asthma [105] and management of asthma according to FeNO levels was effective to reduce maintenance doses of inhaled corticosteroids [106].

Some occupational studies have investigated the role of FeNO in assessing OA, but with inconsistent results [98,102,107-112]. It has been suggested that measurement of FeNO can be used to indicate the development of airway inflammation accompanying late asthmatic reactions after SIC in patients with normal or slightly increased basal NO levels [112]. However, the usefulness of FeNO in the investigation of OA may be limited by factors affecting its determination, such as therapy with inhaled steroids and smoking [100]. Although the measurement of FeNO is totally non-invasive, quick and relatively simple to perform, more data are needed to determine whether FeNO is useful in diagnosing WRA.

• In the clinical setting, a finding of normal exhaled nitric oxide fraction cannot be used to exclude OA

EBC is collected by cooling or freezing exhaled air and is also totally non-invasive [113,114]. In addition to measuring the pH of EBC, investigators have been able to detect several volatile and non-volatile chemicals, including lipids, proteins and oxidants. Nevertheless, there are still open questions in the procedure to collect samples, the reliability of analytical methods for detecting minute concentrations of chemicals and the expression of the results. EBC has been used in the investigation of airway diseases such as asthma, COPD, lung cancer, interstitial lung disease and acute respiratory distress syndrome. Since no studies using EBC have been performed in OA so far, its role in the diagnosis of WRA remains undetermined.

In summary, the above-mentioned new methods are promising for monitoring the inflammatory activity in the airways. However, their definite place in practical diagnosis is at present not clear.

## Diagnosis of diagnostic groups within the spectrum of WRA

Individual diagnostic steps of the following WRA forms correspond to the methods mentioned earlier in the section on diagnosis of allergic WRA. However, SIC may produce positive results in spite of negative IgE findings, e.g. in cases suffering from isocyanate, platinum or irritant asthma with latency (it is clearly inappropriate for acute irritant induced asthma).

1. Work-aggravated asthma. This is an entity characterized by symptomatic deterioration of pre-existing or concomitant non-occupational asthma at work without a latent interval mainly by irritant mechanisms. There are similar changes in lung function related to work exposure as those with allergen- or irritant-induced OA [115].

2. Irritant-induced asthma [116].

a. Acute irritant induced asthma (Reactive airways disease syndrome, RADS) [117]. This is a fairly well described entity. The diagnosis is retrospective and criteria based, e.g. ACCP criteria for diagnosis of reactive airway dysfunction syndrome (RADS) (should meet all eight) [12]:

1. Documented absence of preceding respiratory complaints.

1. Onset of symptoms after a single exposure incident or accident.

1. Exposure to a gas, smoke, fume, or vapor with irritant properties present in very high concentration.

1. Onset of symptoms within 24 h after exposure with persistence of symptoms for at least three months.

1. Symptoms simulated asthma: cough, wheeze, dyspnoea.

1. Presence of airflow obstruction on pulmonary function tests (initial testing should be done shortly after exposure).

1. Presence of nonspecific bronchial hyperresponsiveness.

1. Other pulmonary diseases ruled out.

a. Not so sudden onset of irritant asthma. As opposed to RADS this is caused to lower chronic or repetitive exposures to irritants mostly in the range of the occupational exposure limits [8,118], it is commoner in those with atopy or childhood asthma.

a. Irritant asthma with latency [119]**.** This is a controversial entity of asthma defined by

• ••no prior asthma

• ••a latent interval from first exposure to disease

• ••no massive acute exposure

• ••symptoms related to usual exposure to the causative agent

• ••reproducibility of the asthma from either workplace challenges or specific inhalation challenge or valid serial measurements of PEF measurements at and away from work

• ••an allergic mechanism is very unlikely

## Discussion

### The problem of test materials and lack of standardized technology

Immunological tests are commonly performed as used in allergy testing, but the main problem in occupational allergy is an almost universal lack of standardized requirements for allergy extracts. Simple test may be performed using material that patients bring to the consultation. For HMW agents close collaboration with an immunochemist is advisable for optimal characterization of test material. As regards LMW agents, the problem is even more complex. For instance, there are more than thousands different isocyanates compounds known as yet. In research, most publications deal with monomers such as toluene diisocyanate, hexamethylenediisocyanate, methylene diphenyldiisocyanate. However, some patients may react only with oligomers that are predominantly used by the industry [120,121]. Furthermore, a new generation of isocyanates results from the decomposition of polyurethane coatings or foam during hot work, a process that can yield irritant toxic agents as well [122]. Collaboration with a qualified chemist is thus necessary to plan and monitor tests, especially SIC with complex chemicals.

### Situations with conflicting test results

WRA diagnostic efforts may provide conflicting findings, e.g. the patient may report convincingly work-related asthmatic symptoms but there are no abnormal findings in lung function and allergological tests. Follow-up of the subject at short intervals including serial FEV_1_ and NSBHR monitoring is recommended in such situations. An accelerated FEV_1_ decline can be detected statistically using the Spirola program [123].

## Conclusions

The potential health benefit of a correct and early diagnosis of WRA is considerable both for the individual, industry and society. However, the possibility of WRA is frequently not considered by the doctor and adequate measures to secure an etiologic diagnosis are not performed. Organized collaboration between occupational, primary care and specialized centre physicians is required to improve the situation. An integrated approach is recommended [71].

## Abbreviations

NSBHR: Non-specific bronchial hyperresponsiveness; WRA: Work-related asthma; OA: Occupational asthma; PEF: Peak expiratory flow; SPT: Skin prick test; SIC: Specific inhalation challenge.

## Competing interests

The authors declare that they have no competing interests.

## Authors' contributions

TBAa drafted the manuscript. All co-authors made substantial contributions to the review. All authors read and approved the final manuscript.

## Members of the ERS Task Force on the Management of Work-related Asthma

Xaver Baur, Chair (Hamburg, Bergen Germany); Tor B Aasen (Bergen, Norway); Sherwood Burge (Birmingham, UK); Holger Dressel (Munich, Germany); Dick Heederik (Utrecht, The Netherlands); Paul K Henneberger (Morgantown, USA); PieroMaestrelli (Padova, Italy); Carrie A Redlich (New Haven, USA); Jos Rooijackers (Utrecht, The Netherlands); Vivi Schlünssen (Aarhus, Denmark); Torben Sigsgaard (Aarhus, Denmark); Olivier Vandenplas (Yvoir, Belgium); and Dennis Wilken (Hamburg, Germany).

## References

[B1] Bernstein IL Chan-Yeung M Malo JL Bernstein DI Asthma in the workplace and related conditions 2006 3 New York: Taylor & Francis

[B2] Matte TD Hoffman RE Rosenman KD Stanbury M Surveillance of occupational asthma under the SENSOR model Chest 1990 98 173S 178S 2226005 10.1378/chest.98.5_supplement.173s

[B3] Heederik D Henneberger PK Redlich CA Primary prevention: exposure reduction, skin exposure and respiratory protection Eur Respir Rev 2012 21 112 124 10.1183/09059180.00005111 22654083 PMC9487304

[B4] Wilken D Baur X Barbinova L Preisser A Meijer E Rooyackers J What are the benefits of medical screening and surveillance? Eur Respir Rev 2012 21 105 111 10.1183/09059180.00005011 22654082 PMC9487302

[B5] Beach J Rowe BH Blitz S Crumley E Hooton N Russel K Diagnosis and Management of Work-Related Asthma 2005 Rockville, MD: Agency for Healthcare Research and Quality PMC4780902 16354102

[B6] Nicholson PJ Cullinan P Burge PS Boyle C Occupational asthma: Prevention, identification & management: Systematic review & recommendations 2010 London: British Occupational Health Research Foundation

[B7] Tarlo SM Balmes J Balkissoon R Beach J Beckett W Bernstein D Diagnosis and Management of Work-Related Asthma: American College of Chest Physicians Consensus Statement Chest 2008 134 1S 41S 10.1378/chest.08-0201 18779187

[B8] Baur X Aasen TB Burge PS Heederik D Henneberger PK Maestrelli P The management of work-related asthma guidelines: a broader perspective Eur Respir Rev 2012 21 125 139 10.1183/09059180.00004711 22654084 PMC9487296

[B9] Baur X Sigsgaard T The new guidelines for management of work-related asthma Eur Respir J 2012 39 518 519 10.1183/09031936.00096211 22379145

[B10] Baur X Sigsgaard T Aasen TB Burge PS Heederik D Henneberger P Guidelines for the management of work-related asthma Eur Respir J 2012 39 529 545 10.1183/09031936.00096111 22379148

[B11] Vandenplas O Dressel H Nowak D Jamart J What is the optimal management option for occupational asthma? Eur Respir Rev 2012 21 97 104 10.1183/09059180.00004911 22654081 PMC9487291

[B12] Chan-Yeung M Assessment of asthma in the workplace. ACCP consensus statement. American College of Chest Physicians Chest 1995 108 1084 1117 10.1378/chest.108.4.1084 7555124

[B13] Chan-Yeung M Malo JL Occupational asthma N Engl J Med 1995 333 107 112 10.1056/NEJM199507133330207 7777015

[B14] Global Strategy for Asthma Management and Prevention 2010 Available from http://www.ginasthma.org

[B15] [Anon] National asthma education and prevention program - Expert panel report 3 (EPR-3): Guidelines for the diagnosis and management of asthma - Summary report 2007 J Allergy Clin Immunol 2007 120 S94 S138 17983880 10.1016/j.jaci.2007.09.043

[B16] Hargreave FE Nair P The definition and diagnosis of asthma Clin Exp Allergy 2009 39 1652 1658 10.1111/j.1365-2222.2009.03321.x 19622089

[B17] Hunter CJ Brightling CE Woltmann G Wardlaw AJ Pavord ID A comparison of the validity of different diagnostic tests in adults with asthma Chest 2002 121 1051 1057 10.1378/chest.121.4.1051 11948032

[B18] Gibson PG Simpson JL The overlap syndrome of asthma and COPD: what are its features and how important is it? Thorax 2009 64 728 735 10.1136/thx.2008.108027 19638566

[B19] LindenSmith J Morrison D Deveau C Hernandez P Overdiagnosis of asthma in the community Can Respir J 2004 11 111 116 15045041 10.1155/2004/276493

[B20] Jeebhay MF Quirce S Occupational asthma in the developing and industrialised world: a review Int J Tuberc Lung Dis 2007 11 122 133 17263280

[B21] Sox HC Blatt M Higgins M Marton KI Medical Decision Making 1988 Stoneham, MA: Butterworths

[B22] Johansson SG Bieber T Dahl R Friedmann PS Lanier BQ Lockey RF Revised nomenclature for allergy for global use: Report of the Nomenclature Review Committee of the World Allergy Organization, October 2003 J Allergy Clin Immunol 2004 113 832 836 10.1016/j.jaci.2003.12.591 15131563

[B23] Baur X Bakehe P Vellguth H Bronchial asthma and COPD due to irritants in the workplace - an evidence-based approach J Occup Med Toxicol 2012 7 19 10.1186/1745-6673-7-19 23013890 PMC3508803

[B24] Baur X Bakehe P Allergens causing occupational asthma: an evidence-based evaluation of the literature Int Arch Occup Environ Health 2013 Apr 18. [Epub ahead of print] 10.1007/s00420-013-0866-9 23595938

[B25] Baur X Kay AB, Kaplan AP, Bousquet J, Holt PG Airborne allergens and irritants in the workplace In Allergy and allergic diseases 2008 Oxford: Blackwell Publishing 1017 1022

[B26] Malo JL Ghezzo H d’Aquino C L’Archeveque J Cartier A Chan-Yeung M Natural history of occupational asthma: relevance of type of agent and other factors in the rate of development of symptoms in affected subjects J Allergy Clin Immunol 1992 90 937 944 10.1016/0091-6749(92)90466-F 1460199

[B27] Stenton SC Beach JR Avery AJ Hendrick DJ The value of questionnaires and spirometry in asthma surveillance programmes in the workplace Occup Med (Lond) 1993 43 203 206 10.1093/occmed/43.4.203 8241479

[B28] Vandenplas O Binard-Van CF Brumagne A Caroyer JM Thimpont J Sohy C Occupational asthma in symptomatic workers exposed to natural rubber latex: evaluation of diagnostic procedures J Allergy Clin Immunol 2001 107 542 547 10.1067/mai.2001.113519 11240958

[B29] Baur X Huber H Degens PO Allmers H Ammon J Relation between occupational asthma case history, bronchial methacholine challenge, and specific challenge test in patients with suspected occupational asthma Am J Ind Med 1998 33 114 122 10.1002/(SICI)1097-0274(199802)33:2<114::AID-AJIM3>3.0.CO;2-Y 9438044

[B30] Cote J Kennedy S Chan-Yeung M Quantitative versus qualitative analysis of peak expiratory flow in occupational asthma Thorax 1993 48 48 51 10.1136/thx.48.1.48 8434353 PMC464241

[B31] Cote J Kennedy S Chan-Yeung M Sensitivity and specificity of PC20 and peak expiratory flow rate in cedar asthma J Allergy Clin Immunol 1990 85 592 598 10.1016/0091-6749(90)90098-O 2179365

[B32] Malo JL Ghezzo H L'Archeveque J Lagier F Perrin B Cartier A Is the clinical history a satisfactory means of diagnosing occupational asthma? Am Rev Respir Dis 1991 143 528 532 10.1164/ajrccm/143.3.528 2001062

[B33] Merget R Schultze-Werninghaus G Muthorst T Friedrich W Meier-Sydow J Asthma due to the complex salts of platinum–a cross-sectional survey of workers in a platinum refinery Clin Allergy 1988 18 569 580 10.1111/j.1365-2222.1988.tb02908.x 2468431

[B34] Vandenplas O Delwiche JP Evrard G Aimont P VandB X Jamart J Prevalence of occupational asthma due to latex among hospital personne Am J Respir Crit Care Med 1995 151 54 60 10.1164/ajrccm.151.1.7812572 7812572

[B35] Axon EJ Beach JR Burge PS A comparison of some of the characteristics of patients with occupational and non-occupational asthma Occup Med (Lond) 1995 45 109 111 10.1093/occmed/45.2.109 7718818

[B36] Koskela H Taivainen A Tukiainen H Chan HK Inhalation challenge with bovine dander allergens: who needs it? Chest 2003 124 383 391 10.1378/chest.124.1.383 12853550

[B37] Malo JL Lemiere C Desjardins A Cartier A Prevalence and intensity of rhinoconjunctivitis in subjects with occupational asthma Eur Respir J 1997 10 1513 1515 10.1183/09031936.97.10071513 9230239

[B38] Ricciardi L Fedele R Saitta S Tigano V Mazzeo L Fogliani O Occupational asthma due to exposure to iroko wood dust Ann Allergy Asthma Immunol 2003 91 393 397 10.1016/S1081-1206(10)61687-0 14582819

[B39] American Thoracic Society Guidelines for assessing and managing asthma risk at work, school, and recreation Am J Respir Crit Care Med 2004 169 873 881 15044221 10.1164/rccm.169.7.873

[B40] American Thoracic Society Standardization of spirometry--1987 update. Official statement of American Thoracic Society Respir Care 1987 32 1039 1060 10315742

[B41] Miller MR Hankinson J Brusasco V Burgos F Casaburi R Coates A Standardisation of spirometry Eur Respir J 2005 26 319 338 10.1183/09031936.05.00034805 16055882

[B42] Burge PS O'Brien IM Harries MG Peak flow rate records in the diagnosis of occupational asthma due to colophony Thorax 1979 34 308 316 10.1136/thx.34.3.308 483205 PMC471066

[B43] Park D Moore VC Burge CB Jaakkola MS Robertson AS Burge PS Serial PEF measurement is superior to cross-shift change in diagnosing occupational asthma Eur Respir J 2009 34 574 578 10.1183/09031936.00150108 19324953

[B44] Crapo RO Casaburi R Coates AL Enright PL Hankinson JL Irvin CG Guidelines for methacholine and exercise challenge testing-1999. This official statement of the American Thoracic Society was adopted by the ATS Board of Directors, July 1999 Am J Respir Crit Care Med 2000 161 309 329 10619836 10.1164/ajrccm.161.1.ats11-99

[B45] Sterk PJ Fabbri LM Quanjer PH Cockcroft DW O'Byrne PM Anderson SD Airway responsiveness. Standardized challenge testing with pharmacological, physical and sensitizing stimuli in adults. Report Working Party Standardization of Lung Function Tests, European Community for Steel and Coal. Official Statement of the European Respiratory Society Eur Respir J Suppl 1993 16 53 83 8499055

[B46] Anees W Huggins V Pavord ID Robertson AS Burge PS Occupational asthma due to low molecular weight agents: eosinophilic and non-eosinophilic variants Thorax 2002 57 231 236 10.1136/thorax.57.3.231 11867827 PMC1746281

[B47] Brisman J Lillienberg L Belin L Ahman M Jarvholm B Sensitisation to occupational allergens in bakers' asthma and rhinitis: a case-referent study Int Arch Occup Environ Health 2003 76 167 170 12733091 10.1007/s00420-002-0396-3

[B48] Burge PS Occupational asthma in electronics workers caused by colophony fumes: follow-up of affected workers Thorax 1982 37 348 353 10.1136/thx.37.5.348 7112471 PMC459315

[B49] Cartier A Grammer L Malo JL Lagier F Ghezzo H Harris K Specific serum antibodies against isocyanates: association with occupational asthma J Allergy Clin Immunol 1989 84 507 514 10.1016/0091-6749(89)90364-3 2794294

[B50] Hargreave FE Ramsdale EH Pugsley SO Occupational asthma without bronchial hyperresponsiveness Am Rev Respir Dis 1984 130 513 515 6089628 10.1164/arrd.1984.130.3.513

[B51] Lemiere C Cartier A Malo JL Lehrer SB Persistent specific bronchial reactivity to occupational agents in workers with normal nonspecific bronchial reactivity Am J Respir Crit Care Med 2000 162 976 980 10.1164/ajrccm.162.3.9910031 10988116

[B52] Lin FJ Chen H Chan-Yeung M New method for an occupational dust challenge test Occup Environ Med 1995 52 54 56 10.1136/oem.52.1.54 7697142 PMC1128151

[B53] Merget R Schultze-Werninghaus G Bode F Bergmann EM Zachgo W Meier-Sydow J Quantitative skin prick and bronchial provocation tests with platinum saltMerget Br J Ind Med 1991 48 830 837 1772797 10.1136/oem.48.12.830 PMC1035464

[B54] Merget R Dierkes A Rueckmann A Bergmann EM Schultze-Werninghaus G Absence of relationship between degree of nonspecific and specific bronchial responsiveness in occupational asthma due to platinum salts Eur Respir J 1996 9 211 216 10.1183/09031936.96.09020211 8777953

[B55] Moscato G Dellabianca A Vinci G Candura SM Bossi MC Toluene diisocyanate-induced asthma: clinical findings and bronchial responsiveness studies in 113 exposed subjects with work-related respiratory symptoms J Occup Med 1991 33 720 725 10.1097/00043764-199106000-00014 1650820

[B56] Perrin B Lagier F L'Archeveque J Cartier A Boulet LP Cote J Occupational asthma: validity of monitoring of peak expiratory flow rates and non-allergic bronchial responsiveness as compared to specific inhalation challenge Eur Respir J 1992 5 40 48 1577147

[B57] Tarlo SM Broder I Outcome of assessments for occupational asthma Chest 1991 100 329 335 10.1378/chest.100.2.329 1864102

[B58] Moscato G Godnic-Cvar J Maestrelli P Malo JL Burge PS Coifman R Statement on self-monitoring of peak expiratory flows in the investigation of occupational asthma. Subcommittee on Occupational Allergy of the European Academy of Allergology and Clinical Immunology. American Academy of Allergy and Clinical Immunology. European Respiratory Society. American College of Allergy, Asthma and Immunology Eur Respir J 1995 8 1605 1610 8575590

[B59] Baldwin DR Gannon P Bright P Newton DT Robertson A Venables K Interpretation of occupational peak flow records: level of agreement between expert clinicians and Oasys-2 Thorax 2002 57 860 864 10.1136/thorax.57.10.860 12324671 PMC1746200

[B60] Anees W Gannon PF Huggins V Pantin CF Burge PS Effect of peak expiratory flow data quantity on diagnostic sensitivity and specificity in occupational asthma Eur Respir J 2004 23 730 734 10.1183/09031936.04.00027304 15176688

[B61] Huggins V Anees W Pantin C Burge S Improving the quality of peak flow measurements for the diagnosis of occupational asthma Occup Med (Lond) 2005 55 385 388 10.1093/occmed/kqi072 15860485

[B62] Moore VC Jaakkola MS Burge CB Pantin CF Robertson AS Vellore AD PEF analysis requiring shorter records for occupational asthma diagnosis Occup Med (Lond) 2009 59 413 17 10.1093/occmed/kqp081 19482886

[B63] Leroyer C Perfetti L Trudeau C L'Archeveque J Chan-Yeung M Malo JL Comparison of serial monitoring of peak expiratory flow and FEV1 in the diagnosis of occupational asthma Am J Respir Crit Care Med 1998 158 827 832 10.1164/ajrccm.158.3.9707093 9731012

[B64] Liss GM Tarlo SM Peak expiratory flow rates in possible occupational asthma Chest 1991 100 63 69 10.1378/chest.100.1.63 2060392

[B65] Malo JL Cote J Cartier A Boulet LP L'Archeveque J Chan-Yeung M How many times per day should peak expiratory flow rates be assessed when investigating occupational asthma? Thorax 1993 48 1211 1217 10.1136/thx.48.12.1211 8303625 PMC464971

[B66] Bright P Newton DT Gannon PF Pantin CF Burge PS OASYS-3: improved analysis of serial peak expiratory flow in suspected occupational asthma Monaldi Arch Chest Dis 2001 56 281 288 11665511

[B67] Moore VC Jaakkola MS Burge CB Robertson AS Pantin CF Vellore AD A new diagnostic score for occupational asthma: the area between the curves (ABC score) of peak expiratory flow on days at and away from work Chest 2009 135 307 314002E 10.1378/chest.08-0778 18812450

[B68] Moore VC Jaakkola MC Burge PS A Systematic Review of Serial Peak Expiratory Flow Measurements in the Diagnosis of Occupational Asthma Annals of Respiratory Medicine 2009 1 31 44

[B69] Dreborg S Frew A Allergen standardization and skin tests Allergy 1993 48 49 54 8342740

[B70] Wisnewski AV Developments in laboratory diagnostics for isocyanate asthma Curr Opin Allergy Clin Immunol 2007 7 138 145 10.1097/ACI.0b013e3280895d22 17351466 PMC3131002

[B71] Beach J Russell K Blitz S Hooton N Spooner C Lemiere C A systematic review of the diagnosis of occupational asthma Chest 2007 131 569 578 10.1378/chest.06-0492 17296663

[B72] Budnik LT Preisser AM Permentier H Baur X Is specific IgE antibody analysis feasible for the diagnosis of methylenediphenyl diisocyanate-induced occupational asthma? Int Arch Occup Environ Health 2012 86 417 430 22544379 10.1007/s00420-012-0772-6 PMC3633778

[B73] Platts-Mills TA Longbottom J Edwards J Heymann PW Asthma and rhinitis related to laboratory rats: use of a purified rat urinary allergen to study exposure in laboratories and the human immune response N Engl Reg Allergy Proc 1987 8 245 251 10.2500/108854187779032451 3478580

[B74] Baur X Czuppon A Diagnostic validation of specific IgE antibody concentrations, skin prick testing, and challenge tests in chemical workers with symptoms of sensitivity to different anhydrides J Allergy Clin Immunol 1995 96 489 494 10.1016/S0091-6749(95)70292-X 7560660

[B75] Grammer L Shaughnessy M Kenamore B Utility of antibody in identifying individuals who have or will develop anhydride-induced respiratory disease Chest 1998 114 1199 1202 10.1378/chest.114.4.1199 9792595

[B76] Howe W Venables KM Topping MD Dally MB Hawkins R Law JS Tetrachlorophthalic anhydride asthma: evidence for specific IgE antibody J Allergy Clin Immunol 1983 71 5 11 10.1016/0091-6749(83)90539-0 6822691

[B77] Park HS Kim YJ Lee MK Hong CS Occupational asthma and IgE antibodies to reactive dyes Yonsei Med J 1989 30 298 304 2588667 10.3349/ymj.1989.30.3.298

[B78] Park JW Kim CW Kim KS Choi SY Kang DB Ko SH Role of skin prick test and serological measurement of specific IgE in the diagnosis of occupational asthma resulting from exposure to vinyl sulphone reactive dyes Occup Environ Med 2001 58 411 416 10.1136/oem.58.6.411 11351058 PMC1740145

[B79] Bernstein DI Cartier A Cote J Malo JL Boulet LP Wanner M Diisocyanate antigen-stimulated monocyte chemoattractant protein-1 synthesis has greater test efficiency than specific antibodies for identification of diisocyanate asthma Am J Respir Crit Care Med 2002 166 445 450 10.1164/rccm.2109018 12186818

[B80] Pauli G Soldatov D Kopferschmitt-Kubler MC L'épreuve de provocation bronchiqueest-ellejustifiée en patologierespiratoireprofessionnelle? Rev Pneumol Clin 1996 52 97 102 8761639

[B81] Vandenplas O Malo JL Inhalation challenges with agents causing occupational asthma Eur Respir J 1997 10 2612 2629 10.1183/09031936.97.10112612 9426105

[B82] Ortega HG Weissman DN Carter DL Banks D Use of specific inhalation challenge in the evaluation of workers at risk for occupational asthma: a survey of pulmonary, allergy, and occupational medicine residency training programs in the United States and Canada Chest 2002 121 1323 1328 10.1378/chest.121.4.1323 11948069

[B83] Rioux JP Malo JL L'Archeveque J Rabhi K Labrecque M Workplace specific challenges as a contribution to the diagnosis of occupational asthma Eur Respir J 2008 2008 2008 10.1183/09031936.00100207 18508825

[B84] Pepys J Hutchcroft BJ Bronchial provocation tests in etiologic diagnosis and analysis of asthma Am Rev Respir Dis 1975 112 829 859 173214 10.1164/arrd.1975.112.6.829

[B85] Burge PS Harries MG O'Brien IM Pepys J Respiratory disease in workers exposed to solder flux fumes containing colophony (pine resin) Clin Allergy 1978 8 1 14 10.1111/j.1365-2222.1978.tb00441.x 414855

[B86] Stenton SC Avery AJ Walters EH Hendrick DJ Statistical approaches to the identification of late asthmatic reactions Eur Respir J 1994 7 806 812 10.1183/09031936.94.07040806 8005264

[B87] Burge CB Moore VC Pantin CF Robertson AS Burge PS Diagnosis of occupational asthma from time point differences in serial PEF measurements Thorax 2009 64 1032 1036 10.1136/thx.2009.120923 19850961

[B88] Lemiere C Chaboillez S Malo JL Cartier A Changes in sputum cell counts after exposure to occupational agents: what do they mean? J Allergy Clin Immunol 2001 107 1063 1068 10.1067/mai.2001.115486 11398086

[B89] Djukanovic R Sterk PJ Fahy JV Hargreave FE Standardized methodology of sputum induction and processing Eur Respir J 2002 20 Suppl 37 1 55 12361359 10.1183/09031936.02.00000102

[B90] Maestrelli P Saetta M Di SA Calcagni PG Turato G Ruggieri MP Comparison of leukocyte counts in sputum, bronchial biopsies, and bronchoalveolar lavage Am J Respir Crit Care Med 1995 152 1926 1931 10.1164/ajrccm.152.6.8520757 8520757

[B91] Kips JC Kharitonov SA Barnes PJ Noninvasive assessment of airway inflammation in asthma Eur Respir Mon 2003 23 164 179

[B92] Deykin A Lazarus SC Fahy JV Wechsler ME Boushey HA Chinchilli VM Sputum eosinophil counts predict asthma control after discontinuation of inhaled corticosteroids J Allergy Clin Immunol 2005 115 720 727 10.1016/j.jaci.2004.12.1129 15805990

[B93] Green RH Brightling CE McKenna S Hargadon B Parker D Bradding P Asthma exacerbations and sputum eosinophil counts: a randomised controlled trial Lancet 2002 360 1715 1721 10.1016/S0140-6736(02)11679-5 12480423

[B94] Di Franco A Vagaggini B Bacci E Bartoli ML Cianchetti S Carnevali S Leukocyte counts in hypertonic saline-induced sputum in subjects with occupational asthma Respir Med 1998 92 550 557 10.1016/S0954-6111(98)90307-9 9692121

[B95] Lemiere C Pizzichini MM Balkissoon R Clelland L Efthimiadis A O'Shaughnessy D Diagnosing occupational asthma: use of induced sputum Eur Respir J 1999 13 482 488 10.1183/09031936.99.13348299 10232413

[B96] Maestrelli P Calcagni PG Saetta M Di SA Hosselet JJ Santonastaso A Sputum eosinophilia after asthmatic responses induced by isocyanates in sensitized subjects Clin Exp Allergy 1994 24 29 34 10.1111/j.1365-2222.1994.tb00913.x 8156442

[B97] Lemiere C The use of sputum eosinophils in the evaluation of occupational asthma Curr Opin Allergy Clin Immunol 2004 4 81 85 10.1097/00130832-200404000-00002 15021058

[B98] Obata H Dittrick M Chan H Chan-Yeung M Sputum eosinophils and exhaled nitric oxide during late asthmatic reaction in patients with western red cedar asthma Eur Respir J 1999 13 489 495 10.1183/09031936.99.13348999 10232414

[B99] Quirce S Eosinophilic bronchitis in the workplace Curr Opin Allergy Clin Immunol 2004 4 87 91 10.1097/00130832-200404000-00003 15021059

[B100] ATS/ERS ATS/ERS Recommendations for Standardized Procedures for the Online and Offline Measurement of Exhaled Lower Respiratory Nitric Oxide and Nasal Nitric Oxide, 2005 Am J Respir Crit Care Med 2005 171 912 930 15817806 10.1164/rccm.200406-710ST

[B101] Alving K Weitzberg E Lundberg JM Increased amount of nitric oxide in exhaled air of asthmatics Eur Respir J 1993 6 1368 1370 7507065

[B102] Baur X Barbinova L Latex allergen exposure increases exhaled nitric oxide in symptomatic healthcare workers Eur Respir J 2005 25 309 316 10.1183/09031936.05.00021504 15684296

[B103] Kharitonov SA O'Connor BJ Evans DJ Barnes PJ Allergen-induced late asthmatic reactions are associated with elevation of exhaled nitric oxide Am J Respir Crit Care Med 1995 151 1894 1899 10.1164/ajrccm.151.6.7767537 7767537

[B104] Berlyne GS Parameswaran K Kamada D Efthimiadis A Hargreave FE A comparison of exhaled nitric oxide and induced sputum as markers of airway inflammation J Allergy Clin Immunol 2000 106 638 644 10.1067/mai.2000.109622 11031333

[B105] Smith AD Cowan JO Filsell S McLachlan C Monti-Sheehan G Jackson P Diagnosing asthma: comparisons between exhaled nitric oxide measurements and conventional tests Am J Respir Crit Care Med 2004 169 473 478 10.1164/rccm.200310-1376OC 14644933

[B106] Smith AD Cowan JO Brassett KP Herbison GP Taylor DR Use of exhaled nitric oxide measurements to guide treatment in chronic asthma N Engl J Med 2005 352 2163 2173 10.1056/NEJMoa043596 15914548

[B107] Adisesh LA Kharitonov SA Yates DH Snashell DC Newman-Taylor AJ Barnes PJ Exhaled and nasal nitric oxide is increased in laboratory animal allergy Clin Exp Allergy 1998 28 876 880 10.1046/j.1365-2222.1998.00332.x 9720822

[B108] Allmers H Chen Z Barbinova L Marczynski B Kirschmann V Baur X Challenge from methacholine, natural rubber latex, or 4,4-diphenylmethane diisocyanate in workers with suspected sensitization affects exhaled nitric oxide [change in exhaled NO levels after allergen challenges] Int Arch Occup Environ Health 2000 73 181 186 10.1007/s004200050025 10787133

[B109] Barbinova L Baur X Increase in exhaled nitric oxide (eNO) after work-related isocyanate exposure Int Arch Occup Environ Health 2006 79 387 395 10.1007/s00420-005-0051-x 16421715

[B110] Lund MB Oksne PI Hamre R Kongerud J Increased nitric oxide in exhaled air: an early marker of asthma in non-smoking aluminium potroom workers? Occup Environ Med 2000 57 274 278 10.1136/oem.57.4.274 10810115 PMC1739946

[B111] Olin AC Ljungkvist G Bake B Hagberg S Henriksson L Toren K Exhaled nitric oxide among pulpmill workers reporting gassing incidents involving ozone and chlorine dioxide Eur Respir J 1999 14 828 831 10.1034/j.1399-3003.1999.14d18.x 10573229

[B112] Piipari R Piirila P Keskinen H Tuppurainen M Sovijarvi A Nordman H Exhaled nitric oxide in specific challenge tests to assess occupational asthma Eur Respir J 2002 20 1532 1537 10.1183/09031936.02.00041802 12503715

[B113] Horvath I Hunt J Barnes PJ Alving K Antczak A Baraldi E Exhaled breath condensate: methodological recommendations and unresolved questions Eur Respir J 2005 26 523 548 10.1183/09031936.05.00029705 16135737

[B114] Ferrazzoni S Scarpa MC Guarnieri G Corradi M Mutti A Maestrelli P Exhaled Nitric Oxide and Breath Condensate pH in Asthmatic Reactions Induced by Isocyanates Chest 2009 136 155 162 10.1378/chest.08-2338 19225065

[B115] Chiry S Cartier A Malo JL Tarlo SM Lemiere C Comparison of peak expiratory flow variability between workers with work-exacerbated asthma and occupational asthma Chest 2007 132 483 488 10.1378/chest.07-0460 17505025

[B116] Tarlo SM Workplace respiratory irritants and asthma Occup Med 2000 15 471 484 10769351

[B117] Brooks SM Weiss MA Bernstein IL Reactive airways dysfunction syndrome (RADS) Persistent asthma syndrome after high level irritant exposures. Chest 1985 88 376 384 10.1378/chest.88.3.376 4028848

[B118] Brooks SM Hammad Y Richards I Giovinco-Barbas J Jenkins K The spectrum of irritant-induced asthma: sudden and not-so-sudden onset and the role of allergy Chest 1998 113 42 49 10.1378/chest.113.1.42 9440566

[B119] Burge PS Moore VC Robertson AS Sensitization and irritant-induced occupational asthma with latency are clinically indistinguishable Occup Med (Lond) 2012 62 129 133 10.1093/occmed/kqr211 22199365

[B120] Piirila PL Nordman H Keskinen HM Luukkonen R Salo SP Tuomi TO Long-term follow-up of hexamethylene diisocyanate-, diphenylmethane diisocyanate-, and toluene diisocyanate-induced asthma Am J Respir Crit Care Med 2000 162 516 522 10.1164/ajrccm.162.2.9909026 10934080

[B121] Vandenplas O Cartier A Lesage J Cloutier Y Perreault G Grammer LC Prepolymers of hexamethylene diisocyanate as a cause of occupational asthma J Allergy Clin Immunol 1993 91 850 861 10.1016/0091-6749(93)90342-D 8473673

[B122] Skarping G Dalene M Karlsson D Marand A Dahlin J Adamsson M Air analysis and biomarkers of isocyanates ZentralblArbeitsmedArbeitsschutzProphylErgonomie 2005 55 6 14

[B123] Hnizdo E Yu L Freyder L Attfield M Lefante J Glindmeyer HW Occup Environ Med 2005 62 695 701 10.1136/oem.2004.018424 16169915 PMC1740862

